# Intrapancreatic MSC transplantation facilitates pancreatic islet regeneration

**DOI:** 10.1186/s13287-021-02173-4

**Published:** 2021-02-12

**Authors:** Rahul Khatri, Sebastian Friedrich Petry, Thomas Linn

**Affiliations:** Clinical Research Unit, Centre of Internal Medicine, Faculty of Medicine, Justus Liebig University Giessen, Friedrichstrasse. 20/ Aulweg 123, 35392 Giessen, Germany

**Keywords:** Adipose-derived mesenchymal stem cells, Type 1 diabetes mellitus, Intrapancreatic route, Intravenous route, β cell protection

## Abstract

**Background:**

Type 1 diabetes mellitus (T1D) is characterized by the autoimmune destruction of the pancreatic β cells. The transplantation of mesenchymal stromal/stem cells (MSC) was reported to rescue the damaged pancreatic niche. However, there is an ongoing discussion on whether direct physical contact between MSC and pancreatic islets results in a superior outcome as opposed to indirect effects of soluble factors released from the MSC entrapped in the lung microvasculature after systemic administration. Hence, MSC were studied in direct contact (DC) and indirect contact (IDC) with murine pancreatic β cell line MIN6-cells damaged by nitrosourea derivative streptozotocin (STZ) in vitro. Further, the protective and antidiabetic outcome of MSC transplantation was evaluated through the intrapancreatic route (IPR) and intravenous route (IVR) in STZ-induced diabetic NMRI nude mice.

**Methods:**

MSC were investigated in culture with STZ-damaged MIN6-cells, either under direct contact (DC) or separated through a semi-permeable membrane (IDC). Moreover, multiple low doses of STZ were administered to NMRI nude mice for the induction of hyperglycemia. 0.5 × 10^6^ adipose-derived mesenchymal stem cells (ADMSC) were transferred through direct injection into the pancreas (IPR) or the tail vein (IVR), respectively. Bromodeoxyuridine (BrdU) was injected for the detection of proliferating islet cells in vivo*,* and real-time polymerase chain reaction (RT-PCR) was employed for the measurement of the expression of growth factor and immunomodulatory genes in the murine pancreas and human MSC. Phosphorylation of AKT and ERK was analyzed with Western blotting.

**Results:**

The administration of MSC through IPR ameliorated hyperglycemia in contrast to IVR, STZ, and non-diabetic control in a 30-day window. IPR resulted in a higher number of replicating islet cells, number of islets, islet area, growth factor (EGF), and balancing of the Th1/Th2 response in vivo. Physical contact also provided a superior protection to MIN6-cells from STZ through the AKT and ERK pathway in vitro in comparison with IDC.

**Conclusion:**

Our study suggests that the physical contact between MSC and pancreatic islet cells is required to fully unfold their protective potential.

**Supplementary Information:**

The online version contains supplementary material available at 10.1186/s13287-021-02173-4.

## Background

Type 1 diabetes mellitus (T1D) is an autoimmune disorder manifested by a chronic hyperglycemic state due to insulin deficiency [1]. The worldwide diabetes mellitus prevalence is predicted to grow from 415 million (2015) to 642 million (2040), which poses an enormous economic and financial burden [2]. Out of which, T1D contributes to 10–15% especially children below the age of 2 years [3]. Since uncontrolled blood glucose will lead to both macrovascular and microvascular diabetic complications [4], patients are required to maintain their blood glucose level within a narrow range. However, multiple daily injections of exogenous insulin as the standard therapeutic practice entail recurrent hypoglycemic episodes and restricted life quality.

In recent years, MSC-based remedies have emerged as a persuasive source of T1D treatment including the prevention of its secondary complications and β cell substitution [5–7]. MSC are non-hematopoietic, fibroblast-like, multipotent stromal cells which could be gleaned efficiently from a wide variety of tissues and rapidly undergo mesodermal lineage differentiation, for instance, cardiomyocytes, myoblasts, adipocytes, chondrocytes, and β cell-like cells [8–11]. MSC T1D therapy was reported to ameliorate hyperglycemia, stimulate pancreatic islet repair mechanisms by secreting cytokines and growth factors, and immunomodulate the host’s immune system [12, 13].

MSC administered through a systemic route are exposed to lung microvasculature entrapment, the sequel in restricted access to the target organ, and display inferior protective potential [14–17]. In a randomized trial with T1D patients, a single intravenous injection prevented C-peptide loss up to 1 year but failed to regulate blood glucose levels without an additional insulin application [14], while the intravenous infusion of human umbilical cord blood-derived MSC and the intrapancreatic injection were nearly equivalent in balancing the blood glucose level in diabetic mice [15]. However, in some reports, the intrapancreatic route of MSC application resulted in a superior curative effect on blood glucose concentrations compared to the intravenous administration [16–18]. MSC administered through the intrapancreatic route reversed the hyperglycemic state in 42% and via the intrasplenic route in 70% of diabetic recipient mice [19]. As possible underlying mechanisms, Murai et al. demonstrated a reduced number of activated macrophages in the murine pancreas after intrapancreatic injection of human BM-MSC and confirmed the intrapancreatic supremacy over intravenous injection in T1D [20].

Taken together, both routes of administration seem to exert restorative effects on damaged β cells, but with differentially activated patterns [21–24]. As a major difference, the intrapancreatic route (IPR) of transplantation allows the physical contact of MSC with pancreatic β cells, while the intravenous route (IVR) leads to their off-site accumulation and only indirect effects on the pancreas. However, it is still uncertain whether the physical contact between MSC and pancreatic β cells is vital for a better outcome, or a distant effect is sufficient for MSC-based therapies aiming for β cell preservation. Moreover, the source of the MSC from different human tissues might influence the therapeutic efficacy. Therefore, we utilized the immortalized bone marrow-derived hTERT-MSC line to study the interaction of MSC with β cells in co-culture system. In addition, primary MSC from adipose tissue were applied for the in vivo transfer into diabetic NMRI nude mice via IVR and IPR routes. Our study provides evidence for a superior antidiabetic effect of locally administered MSC over the systemic route in STZ-induced diabetic NMRI nude mice, which might be beneficial for future therapies focusing on β cell preservation and restoration.

## Material and methods

### Cell culture

#### MIN6-cells

MIN6-cells are defined as a β cell line that originated from mouse insulinoma, 6th subclone. MIN6-cells were cultivated and maintained in DMEM (Gibco, Germany) with 50 μM β-mercaptoethanol (Sigma, Germany), 20% FBS (Biowest, USA), and 1% penicillin-streptomycin in an incubator with 5% CO_2_ at 37 °C.

#### hTERT-MSC

hTERT-MSC were seeded with 5 × 10^4^ cells/cm^2^ in MEM media (Gibco, Germany) with 1% l-glutamine (Invitrogen, Germany), 10% FBS (Biowest, USA), and 1% penicillin-streptomycin (Invitrogen, Germany) and maintained at 37 °C in an incubator. After attaining 70–80% confluency, hTERT-MSC were employed for further experiments.

#### Adipose-derived mesenchymal stem cells (ADMSC)

ADMSC were isolated as detailed previously [25]. Briefly, ADMSC were seeded with 5 × 10^4^ cells/cm^2^ in T-175 flask (Corning, USA) in DMEM media (D5671, Sigma) with 20% FBS (Biowest, USA), 1% non-essential amino acid (NEAA, Gibco, Germany), 1% l-glutamine (Invitrogen, Germany), and 1% penicillin-streptomycin (Invitrogen, Germany) with 5% CO_2_ at 37 °C in an incubator. For the transplantation, cells were detached with trypsin-EDTA. The enzymatic reaction was halted with 20% FBS containing DMEM media. Cells were counted with trypan blue in a Neubauer chamber, and 0.5 × 10^6^/100 μL was considered for the transplantation.

### Direct contact (DC) and indirect contact (IDC) co-culture system

#### Viability

Five thousand MIN6-cells/well were seeded overnight in a 96-well plate for DC. MIN6-cells were incubated with STZ (0.5 mM, 1.0 mM, or 2.0 mM) for another 24 h to induce injury. Further, 2000 hTERT-MSC were added to attain physical contact for 24 h. In the case of IDC, 5000 MIN6-cells/well were subjected to HTS Transwell™ 96-Well Permeable Support System, Corning™ overnight, and incubated for 24 h with STZ (0.5 mM, 1.0 mM, or 2.0 mM). The next day, 2000 hTERT-MSC were grown on a transwell with pore size 0.4 μm for 24 h. Ninety-six-well plate from DC and IDC was continued with MTT (50 μL from the stock solution of 2 mg/mL, Sigma, Germany) and maintained in dark for 4 h at 37 °C. Further, 200 μL DMSO was added and incubated for another 1 h at RT. Mithras LB 940 Multimode Microplate Reader was adopted to capture the absorbance at 590 nm and 620 nm (reference filter).

#### Migration assay

In 24-well plate, 0.3 × 10^6^ MIN6-cells were grown overnight and challenged with STZ (0.5 mM, 1.0 mM, or 2.0 mM) for 24 h. On the following day, 8-μm pore size inserts including 2 × 10^4^ hTERT-MSC were added to each well. After 24 h, inserts were taken, and the inner portion was cleaned with cotton. The membrane was washed and developed with the FDA for 15 min at 37 °C. Migrated hTERT-MSC were assessed after capturing pictures and analyzing them with ImageJ software (National Institutes of Health, USA).

### Induction of diabetes and transfer of ADMSC to mice

NMRI nu/nu athymic mice with the age of 10 to 12 weeks were acquired from Janvier (France). Mice were retained at a 12:12-h cycle (light to dark) with 24 ± 2 °C temperature and equipped with standard food (1324 TFP, ad libitum, Altromin, Germany) and water. All procedures were pursued in conformity with the German Animal Welfare Law and institutional guidelines (code 31/2017).

In this experiment, four groups were established: control (sham control; non-diabetic and non-transplanted mice), STZ, IVR, and IPR. Three groups (STZ, IVR, and IPR) were injected with multiple low doses of streptozotocin (STZ) intraperitoneally (Fig. 3a). Forty milligrams per kilogram body weight of STZ was administered for three successive days culminating in an overall dose of 120 mg/kg body weight. Blood glucose was tested with a hand-held glucose meter (One Touch® Ultra®2, LifeScan) by pin-pricking the tail vein.

Mice with a blood glucose level above 11.1 mmol/L were considered for transplantation. They were anesthetized with xylazine (20 mg/kg body weight; Ceva, Germany) and ketamine (100 mg/kg body weight; Medistar, Germany). A middle line incision was accomplished to uncover the pancreas. In the IPR group, 0.5 × 10^6^/100 μL ADMSC were injected gently into the intermediate part of the pancreas (3–5 mm deep) accompanied by the closure of the abdominal skins with synthetic absorbable suture (Vicryl suture 5-0, Ethicon, USA) within 10–15 min. The IVR group received ADMSC (0.5 × 10^6^/100 μL) through a tail vein. At the end of the experiment, BrdU (100 mg/kg body weight) was administered for three consecutive days before the organ retrieval as shown in Fig. 3a.

### RT-PCR

Total RNA from the mouse pancreas was extracted with peqGOLD TrifastTM reagent (peqlab, Germany) and pursued with RNeasy Mini Kit (Qiagen). However, RNeasy Micro Kit (Qiagen) was employed for the hTERT-MSC and MIN6-cell RNA isolation as per the manufacture’s instruction. c-DNA was transcribed with 1 μg RNA by utilizing the SuperScript III Reverse Transcriptase kit (Invitrogen, Germany). RT-PCR (StepOnePlus, Applied Biosystems) was accomplished with SYBR Green Supermix (Bio-Rad Laboratory, Germany), c-DNA template, and primers (20 pM). PCR reaction was carried out with initial denaturation at 95 °C for 10 min, denaturation (95 °C, 15 s) for 40 cycles, annealing at 60 °C for 30 min, extension (60 °C, 1 min), and melting curve. For the primer list, refer to Table 1.

**Table 1:** RT-PCR primers

Primer	Forward primer	Reverse primer
m EGF	5′-TCTCGGATTGACC CAGAT-3′	5′-CCCAGACACCTTCCTCTCT-3′
m Ins1	5′-TATAAAGCTGGTGGGCATCC-3′	5′-GGGACCACAAAGATGCTG TT-3′
m Ins2	5′-GGCTTCTTCTACACACCCATGT-3′	5′-AAGGTCTGAAGGTCACCTGCTC-3′
m FoxO1	5′-TTCAATTCGCCACAATCTG TCC-3′	5′-GGGTGATTTTCCGCTCTTGC-3′
m TNF-α	5′-CATCTTCTCAAAATTCGAGTGACAA-3′	5′-TGGGAGTAGACAAGG TACAACCC-3′
m SDF-1	5′-AACCAGTCAGCCTGAGCTAC-3′	5′-GGGTCAATGCACACTTGTCTG-3′
m BCL2	5′-ATGCCTTTGTGGAACTATATGGC-3′	5′-GGTATGCACCCAGAGTGATGC-3′
m BAX	5′-TGAAGACAGGGGCCTTTTTG-3′	5′-AATTCGCCGGAGACACTCG-3′
m IL-1β	5′-AGGTCGCTCAGGGTCACAAG-3′	5′-GTGCTGCCTAATGTCCCCTTGAATC-3′
m IL-10	5′-TAAGGCTGGCCACACTTGAG-3′	5′-GTTTTCAGGGATGAAGCGGC-3′
m ERK	5′-TCAGTTTGTCCCCTTCCATTG-3′	5′-TCCACTCCCACAATGCACAC-3′
m DLK1	5′-AGTGCGAAACCTGGGTGTC-3′	5′-GCCTCCTTGTTGAAAGTGGTCA-3′
m RPL32	5′-GGAGAAGGTTCAAGGGCCAG-3′	5′-GCGTTGGG ATTGGTGACTCT-3′
h CXCR4	5′-GACTGGCATAGTCGGCAATG-3′	5′-AGAAGGGGAGTGTGATGACAAA-3
h TIMP1	5′-TTGTGGACGGACCAGCTCCT-3′	5′-GGTGGACACTGTGCAGGCTT-3′
h IDO1	5′-AGCTGCGCTGATAGACATCC-3′	5′-GGCGCTGTGACTTGTGGTCT-3′
h VEGF	5′-CTACCTCCACCATGCCAAGT-3′	5′-AGCTGCGCTGATAGACATCC-3′
h RPL13	5′-CCTGGAGGAGAAGAGGAAAGAGA-3′	5′-TTGAGGACCTCTGTGTATTTGTCAA-3′

### Western blot

MIN6-cell lysates were produced with 1X RIPA buffer (200 μL; Cell Signalling Technology, Germany) possessing protease inhibitor (Thermo Scientific, Germany). Lysates were vortexed and incubated for 20 min on ice. After centrifugation (12,000 rpm at 4 °C for 20 min), the protein was collected and quantified with Bio-Rad Protein Assay (Bio-Rad, Germany). Sodium dodecyl sulfate-polyacrylamide gel electrophoresis (SDS-PAGE) was implemented, and proteins were transferred on activated polyvinylidene fluoride (PVDF; EMD) followed by blocking with 5% milk powder (Sigma, Germany). The membrane was exposed to primary antibodies: AKT (1:1000, Rabbit AKT Antibody, Cell signaling), p-AKT (1:1000, Rabbit Phospho-AKT; Ser473, Cell signaling), ERK (1:1000, Rabbit ERK Antibody, Cell signaling), and p-ERK (1:1000, Rabbit Phospho-p44/42 MAPK; Erk1/2, Cell signaling) overnight at 4 °C. The membrane was rinsed with TBST thrice and incubated with Polyclonal Goat Anti-Rabbit Immunoglobulins/HRP secondary antibody (1:3000; Dako, Germany) and pursued with enhanced chemiluminescence (ECL) developing system.

### Immunohistochemistry

After 30 days, the pancreases were retrieved from all four groups and fixed with 4% paraformaldehyde for 6 h followed by washing and paraffin embedding. Pancreatic sections with 5- to 7-μm thickness were cut and processed for antigen retrieval. Sections were exposed to NaOH, processed further for blocking with goat serum (1%) at RT for 20 min, stained with primary antibody: polyclonal guinea pig anti-insulin, DAKO (1:100 from the stock of 26.1 g/L) overnight at 4 °C, and afterward subjected to secondary antibodies: AP-conjugated affinity-purified anti-guinea pig from Rockland, Germany (1:40 from the stock of 1 mg/mL) for 1 h at RT. Insulin was developed with a vector blue substrate kit (Vector Laboratory, Germany). Further, a rodent blocker (BIOCARE MEDICAL, Germany) was administered for 30 min before the treatment with an anti-BrdU antibody (1:100 from the stock of 262 mg/mL, DAKO, Germany) at 4 °C overnight. A mouse on mouse HRP polymer (BioCare Medical, Germany) was employed, and the BrdU-positive brown color was developed with ImmPACT™ AMEC Red Substrate. A light microscope (Leica microsystem, ICC50 HD) was utilized to capture the pictures. Morphometric analyses were standardized using blinded examiner readings.

### ELISA

After 30 days, blood serum and pancreas were isolated from all the four groups. The pancreas was mechanically grinded and exposed to acid ethanol as reported earlier [26, 27]. Blood was centrifuged at 3000 rpm for 10 min at 4 °C, and serum was collected. A mouse Insulin ELISA kit (DRG Instruments GmbH, Germany) was used to measure the insulin content.

### Statistical analysis

GraphPad Prism 8 (GraphPad, USA) was employed for statistics. Data were shown as a mean ± SEM. Multiple *t* test or two-way ANOVA was adopted with post hoc analysis depending on the experiments unless otherwise stated. For the survival curve, the log-rank (Mantel-Cox) test was applied. Significant statistics are illustrated by *p ≤ 0.05, **p ≤ 0.01, and ***p ≤ 0.001.

## Results

### hTERT-MSC protect MIN6-cells from STZ-mediated injury

**Fig. 1:**
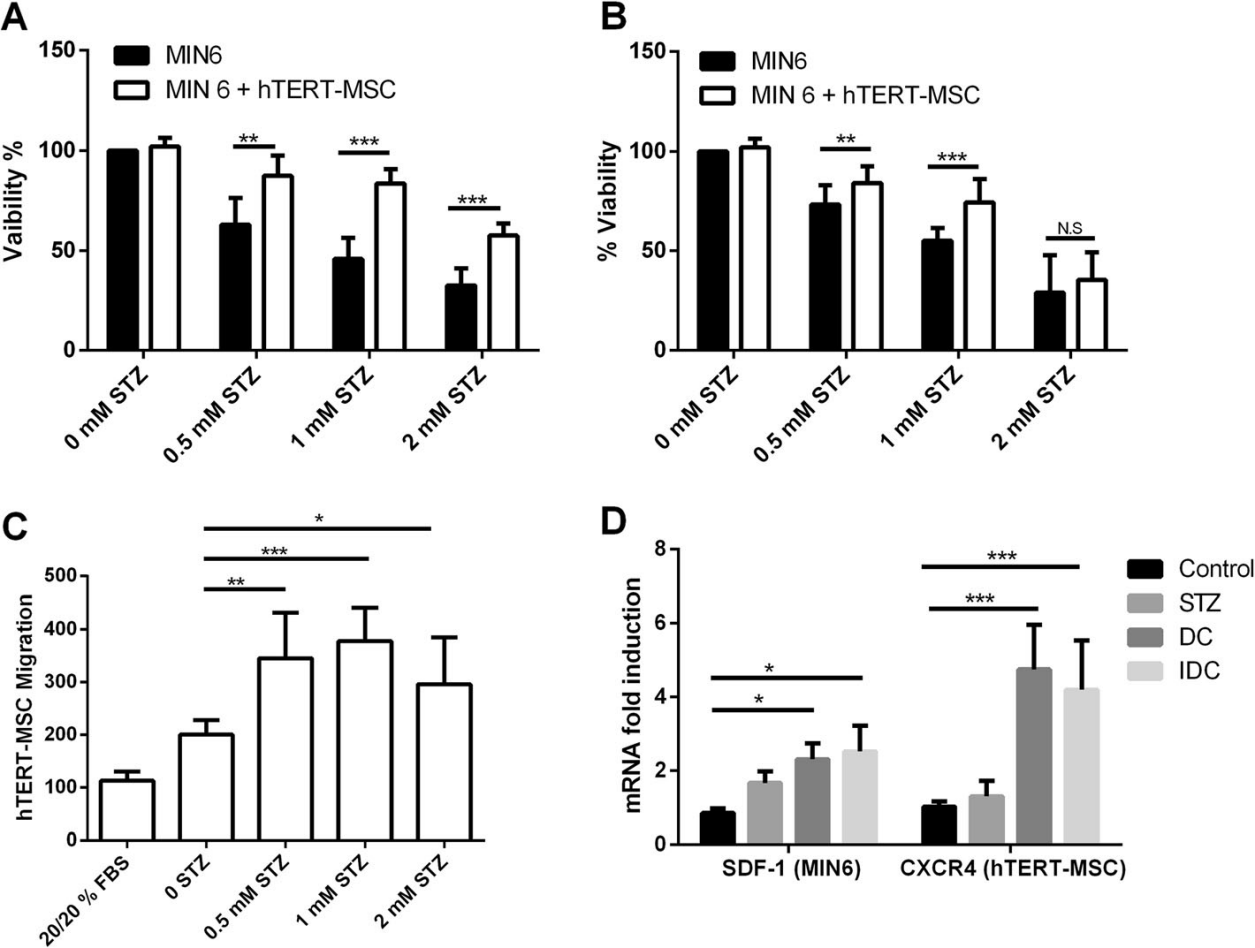
hTERT-MSC migrate to damaged MIN6-cells and protect them from STZ toxicity. After treatment of MIN6-cells with 0.5, 1.0, or 2.0 mM STZ for 24 h, hTERT-MSC were added to attain direct contact (DC) for 24 h. For studying indirect paracrine effects (IDC), MIN6-cells were subjected to a transwell 96-well plate overnight and incubated for 24 h with STZ. On the next day, hTERT-MSC were added for 24 h to inserts of the wells separating them from MIN6-cells by a membrane with a pore size of 0.4 μm to allow for diffusion of soluble factors only. The influence of hTERT-MSC on the viability of MIN6-cells in DC (**a**) and IDC (**b**) at three concentrations of STZ (0.5, 1.0, and 2.0 mM) with MTT assay is demonstrated. The MIN6-cells’ viability expressed is depicted in the percentage of untreated controls in the presence (STZ) and absence (control) of STZ with their respective controls. Approximately 60% viability was noticed in DC which reduced to 35% in IDC at 2.0 mM. **c** hTERT-MSC migration was evaluated on the membrane of the Boyden chamber with FDA staining in IDC. Unspecific background migration of hTERT-MSC was neutralized by considering 20% FBS in both the chamber (Boyden chamber and well). **d** RT-PCR was employed to identify the expression of murine SDF-1 in MIN6-cells and human CXCR4 in hTERT-MSC. IDC, indirect culture; DC, direct culture; hTERT-MSC, human telomerase reverse transcriptase mesenchymal stem cells; CXCR-4, C-X-C chemokine receptor type 4; SDF1, stromal cell-derived factor 1. Data represent the mean ± SEM, *n* = 4. Value considered significant at *p* ≤ 0.05, **p ≤ 0.01, and ***p ≤ 0.001

Mesenchymal stem cells (MSC) are proposed to provide protection from organ damage by the release of growth factors and anti-inflammatory molecules. The effect is regarded as being dependent on the cell’s tissue source and the distance of the MSC to the site of injury. Therefore, the protective outcome of hTERT-MSC on STZ-injured MIN6-cells was examined comparing, first, direct cellular connection (DC) and, second, cells separated by an 8-μm membrane that maintained the effect of molecules released in the medium (IDC). hTERT-MSC rescued the viability of about one-third of adjacent (DC) MIN6-cells damaged by rising STZ concentrations [0.5 mM (MIN6 63 ± 13.3% vs MIN6 + hTERT-MSC 87.4 ± 10.2%), 1.0 mM (MIN6 45.8 ± 10.4% vs MIN6 + hTERT-MSC 83.6 ± 7.2%), 2.0 mM (MIN6 32.6 ± 8.5% vs MIN6 + hTERT-MSC 57.6 ± 6%)] as shown in Fig. 1a. hTERT-MSC also improved the viability of co-cultured MIN6-cells under IDC conditions at 0.5 mM (MIN6 73.4 ± 9.6% vs MIN6 + hTERT-MSC 84.2 ± 8.4%) and 1.0 mM (MIN6 55.4 ± 6.4% vs MIN6 + hTERT-MSC 74.4 ± 11.7%), but were incompetent to provide protection at 2.0 mM (MIN6 29.1 ± 18.8% vs MIN6 + hTERT-MSC 35.4 ± 13.9%) concentration of STZ (Fig. 1b).

Next, cell migration towards STZ-injured MIN6-cells was examined. The directed movement of hTERT-MSC was triggered by all three STZ dosages (0.5 mM: *p* ≤ 0.01, 1.0 mM, *p* ≤ 0.001; and at 2.0 mM STZ: *p* ≤ 0.039; Fig. 1c). Interestingly, chemotactic factor SDF-1 gene expression was induced in MIN6-cells in both co-culture systems (IDC: *p* ≤ 0.026 and DC: *p* ≤ 0.041). CXCR4 gene encoding the receptor specific for SDF-1 was expressed at both IDC (*p* ≤ 0.001) and DC (*p* ≤ 0.001) in hTERT-MSC (Fig. 1d).

**Fig. 2:**
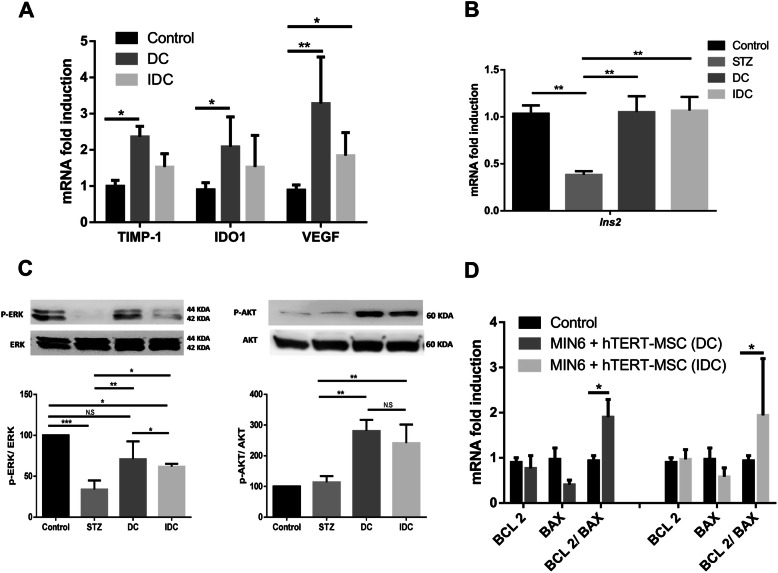
Protective pathways activated in MIN6-cells by hTERT-MSC. **a** hTERT-MSC manufactured VEGF, IDO1, and TIMP-1 after interacting with STZ-injured MIN6-cells in DC and IDC. Comparatively, higher expression of these molecules were observed in DC. Control is considered as healthy hTERT-MSC without physical or indirect contact with MIN6-cells. **b** The presence of hTERT-MSC in both conditions DC and IDC restored *Ins2* gene expression in STZ-injured MIN6-cells. Control is considered as healthy MIN6-cells without STZ and hTERT-MSC influence. **c** The phosphorylation of AKT and ERK in damaged MIN6-cells with representative nitrocellulose membrane in upper and quantification of expressions in the lower panel with control adjusted to a hundred percent. A similar amount of phosphorylated AKT was detected in DC (280.8 ± 36.40) and IDC (241.4 ± 50.29; *p* ≤ 0.341), but the higher p-ERK expression was noticed in DC (75.93 ± 8.2) compared to IDC (59.18 ± 2.4; *p* ≤ 0.05). **d** Next, we hypothesized that p-AKT and p-ERK stimulated by hTERT-MSC influenced BCL-2 and BAX signaling cascade. In fact, MIN6-cells showed an increased BCL2/BAX ratio in the presence of MSC indicating a cellular state of anti-apoptosis. The four experimental conditions were identical to those described in Fig. 1. IDC, indirect culture; DC, direct culture; hTERT-MSC, human telomerase reverse transcriptase mesenchymal stem cells; IDO1, indoleamine 2,3-dioxygenase 1; TIMP-1, TIMP metallopeptidase inhibitor 1; VEGF, vascular endothelial growth factor; *Ins2*, preproinsulin 2; ERK, extracellular signal-regulated kinases; AKT, protein kinase B; BCL-2, B cell lymphoma 2; BAX, BCL-2-associated X protein. Data represent the mean ± SEM, *n* = 4. Value considered significant at *p* ≤ 0.05, **p ≤ 0.01, and ***p ≤ 0.001

Further, hTERT-MSC in direct or indirect co-culture with damaged MIN6-cells enhanced the synthesis of VEGF transcripts compared to MSC cultivated in the absence of MIN6-cells (Fig. 2a, DC (3.2 ± 1.2), *p* ≤ 0.01 and IDC (1.8 ± 0.6), *p* ≤ 0.035). Similarly, transcripts of immunomodulatory factors TIMP-1 and IDO1 were statistically upregulated only in DC [TIMP-1 (2.3 ± 0.2), *p* ≤ 0.049; IDO1 (2.08 ± 0.8), *p* ≤ 0.04] but failed in IDC [TIMP-1 (1.5 ± 0.3), *p* ≤ 0.068; IDO1 (1.49 ± 0.8), *p* ≤ 0.37] (Fig. 2a). The presence of hTERT-MSC preserved *Ins2* expression in STZ-injured MIN6-cells (DC, *p* ≤ 0.006; IDC, *p* ≤ 0.0062) in contrast to STZ-injured MIN6-cells only as shown in Fig. 2b.

The impact of hTERT-MSC on pathways crucial for insulin was further explored in MIN6-cells with Western blot. At 1.0-mM STZ treatment, phosphorylation of AKT protein (p-AKT) was noticed in MIN6-cells in DC (*p* ≤ 0.0023) and IDC (*p* ≤ 0.0064) compared to STZ (Fig. 2c). Interestingly, higher phosphorylation of ERK protein (p-ERK) was observed in DC than IDC (*p* ≤ 0.035) and STZ (*p* ≤ 0.01; Fig. 2c). Further, the ratio of BCL-2 versus BAX was measured in both IDC (*p* ≤ 0.0428) and DC (*p* ≤ 0.0389) as illustrated in Fig. 2d.

### Superior glycemic effect of intrapancreatic versus intravenous transfer of ADMSC

**Fig. 3:**
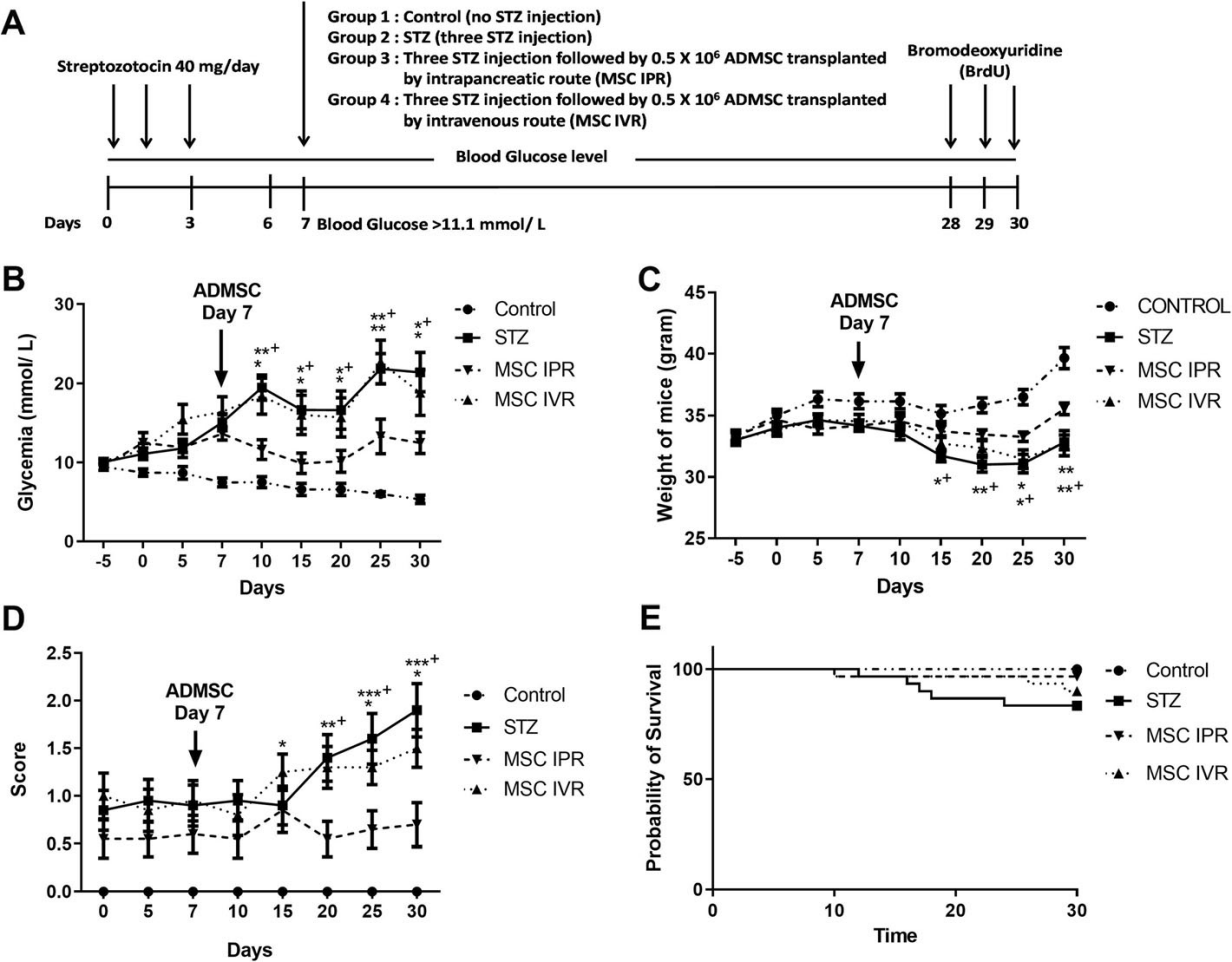
Effect of ADMSC administration in diabetic NMRI nude mice. **a** Systematic representation of the experimental design. Three doses of STZ were injected to render the mice diabetic. After attaining blood glucose level above 11.1 mmol/L on day 7, ADMSC were administered with IPR and IVR. **a** Four experimental groups (*n* = 11 mice each) are represented in **a**; control is non-diabetic and non-transplanted mice; STZ is STZ-induced diabetic mice without ADMSC treatment; MSC-IVR is STZ-induced diabetic mice transplanted with ADMSC by intravenous route; MSC-IPR is STZ-induced diabetic mice transplanted with ADMSC by the intrapancreatic route. **b** The improved blood glucose profile after local transplantation of ADMSC (IPR) compared to the systemic route and STZ only group. **c** Body weight was measured throughout the experiment. At day 15, substantial variations were observed in the mean body weight of various groups. **d** The health score calculated from a list of health indicators designed for T1D (Table 2) in distinct groups. The IPR group exhibited a lower score as contrasted to others. **e** Survival rate remained insignificant for up to 30 days with a tendency of best survival in IPR as opposed to the IVR group. ADMSC, adipose tissue-derived mesenchymal stem cells; STZ, streptozotocin; IPR, intrapancreatic route; IVR, intravenous route; no STZ and no MSC, control. Data represent the mean ± SEM, *n* = 11. Value considered significant at *p* < 0.05, **p ≤ 0.01, and ***p ≤ 0.001. *^+^Comparison between IPR and STZ and *comparison between IPR and IVR

To study the distinctive effects of local and systemic MSC transplantation in vivo, four experimental groups were followed, out of which IVR and IPR were transplanted with ADMSC (Fig. 3a). The overall non-fasting blood glucose concentration in NMRI nu/nu mice during the 30-day follow-up was 8.7 ± 1.8 mmol/L. The injection of STZ resulted in blood glucose levels up to 19.5 ± 5.3 mmol/L in mice without MSC treatment on day 10. ADMSC transplanted on day 7 decreased the mean blood glucose after 3 days in the IPR group (11.5 ± 4.1 mmol/L; day 10) compared to STZ only (19.5 ± 4.3 mmol/L, *p* ≤ 0.0162; day 10) and IVR (18.4 ± 5.4 mmol/L, *p* ≤ 0.003; day 10) as indicated in Fig. 3b. Throughout the follow-up till 30 days, the IPR group maintained lower mean blood glucose values (12 ± 4.4 mmol/L; day 30) as contrasted to STZ alone (21.4 ± 8.2 mmol/L, *p* ≤ 0.0238; day 30), IVR (18.8 ± 8.1 mmol/L; *p* ≤ 0.0373; day 30), and the non-diabetic control (6.6 ± 1.3 mmol/L, *p* ≤ 0.0426; day 30) (Fig. 3b).

Analogously, body weight varied from 33.7 ± 1.2 g (day minus five) to day 15 in the IVR (32 ± 2.1 g), IPR (33.7 ± 1.6 g), STZ (31 ± 1.6 g), and non-diabetic control (35 ± 1.7 g). On day 30, a statistical significance was detected in IPR (35 ± 1.57 g) compared to IVR (32.75 ± 2.91 g, *p* ≤ 0.005), STZ (32.8 ± 1.99 g, *p* ≤ 0.0024), and control (39 ± 2.06 g, *p* ≤ 0.001) as presented in Fig. 3c. Health scores summarized from items listed in Table 2 exhibited a significant variation in the IPR versus STZ (*p* ≤ 0.001) and IVR (*p* ≤ 0.014) groups (Fig. 3d). The highest survival was displayed by the non-diabetic control (100%) accompanied by IPR (96.6%), IVR (90%), and STZ (83.3%) at the end of the experiment without achieving significance (Fig. 3e).

**Table 2:** Score sheet

**Mouse identification:**	**Date:**								
	**Time:**								
**1. Body weight**	**Score**								
Based on starting weight []									
Based on the weight of the control group []									
Uninfluenced or rise	**0**								
Reduction, but < 10%	**1**								
Reduction > 10%	**2**								
Reduction > 20%	**3**								
**2. General condition**									
Shiny eyes, body openings and skin clean	**0**								
Cloudy eyes, increased muscle tone, more visible breathing	**1**								
Eyes sunken dull, sticky body openings, increased breathing	**2**								
Abnormal posture, animal feels cold, eyes closed, cramps, paralysis, breathing sounds, bluish mucous membranes, diarrhoea	**3**								
**3. Spontaneous behaviour**									
Attentive, curious, straightening, quick movements	**0**								
Decreased reactions, movement reduced, restricted or excessive activity	**1**								
Partial separation from the group, movement reduced, pain when walking	**2**								
Apathetic, no reaction or aggressiveness in handling, severely restricted movement, isolation, drag forward.	**3**								
**4. Trial-specific criteria**									
Blood sugar < 200 mg/dl	**0**								
Blood sugar increased (> 200 mg/dl)	**1**								
Blood sugar increased (> 400 mg/dl)	**2**								
Blood sugar reduced (< 60 mg/dl) At the same time, weight loss	**3**								
**5. Other termination criteria**									
Self-injury (e.g. excessive itching)	**3**								
**Total score**									

### Secure injection of ADMSC to the diabetic pancreas

**Fig. 4:**
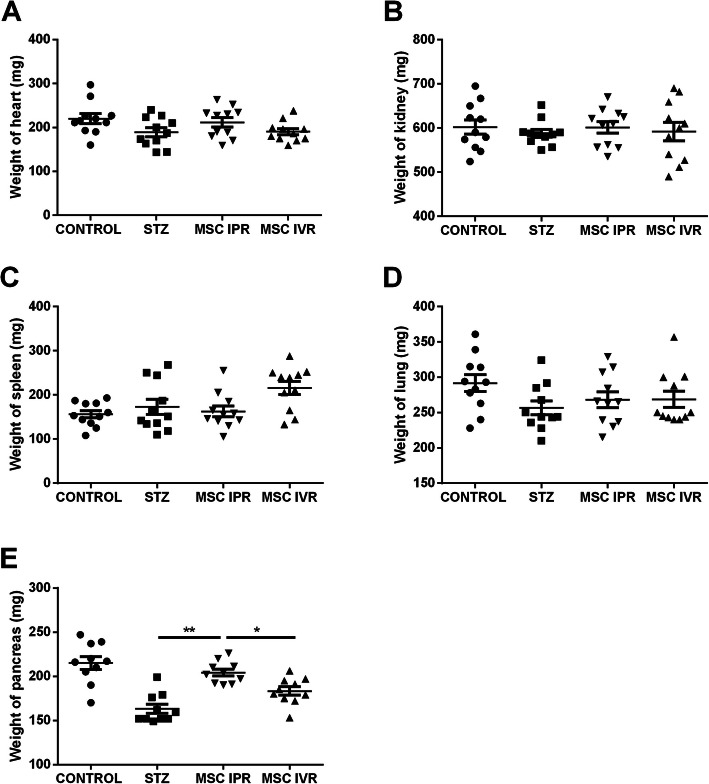
ADMSC were administered in STZ-induced diabetic mice through IVR and IPR. At day 30, organs were retrieved and weighed. **a**–**d** The weights of the heart, kidney, spleen, and lung. No variations were assessed among groups. **e** A considerable reduction of mean pancreatic weight was noted in STZ and IVR groups compared to IPR. Interestingly, no significant difference was detected between control and IPR body weights. STZ, streptozotocin; IPR, intrapancreatic route; IVR, intravenous route; no STZ and no MSC, control. Data represent the mean ± SEM, *n* = 11. Value considered significant at *p* ≤ 0.05, **p ≤ 0.01, and ***p ≤ 0.001

STZ was administered for three consecutive days followed by the transplantation of MSC via the above-mentioned routes. At day 30, vital organs such as the pancreas, spleen, heart, kidney, and lung were retrieved and weighed. Considerable variation was noticed in the pancreas weight (204 ± 3.92 mg) in IPR as opposed to STZ only (163 ± 5.18 mg, *p* ≤ 0.0012) and IVR (183 ± 4.76 mg, *p* ≤ 0.047) but not to the non-diabetic control group (215 ± 7.37 mg, *p* ≤ 0.50) (Fig. 4e) indicating that the injection procedure of ADMSC into the diabetic pancreas was reasonably atraumatic. The other organ weights remained unchanged during the observation time (Fig. 4a–d). After transplantation, ADMSC were tracked with the human Alu sequence. In IVR, human DNA was noticed in the lung (1/10), kidney (1/10), and pancreas (1/10) whereas, in the IPR group, human DNA was encountered only in the pancreas (2/10) at the end of the experiment (Supplement Figure S1).

### Pancreatic islet cell proliferation

**Fig. 5:**
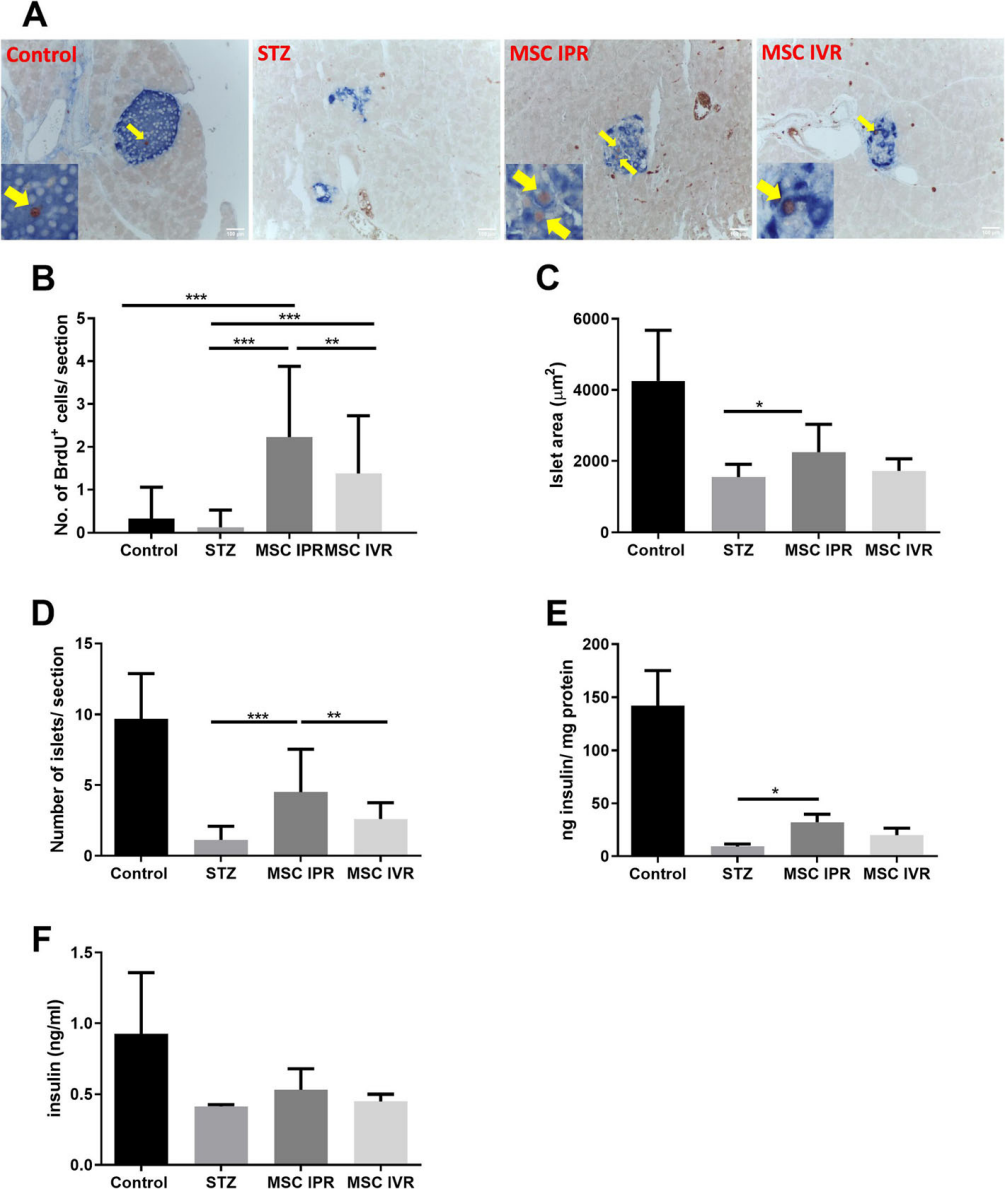
The morphometry of pancreatic islets after ADMSC administration in STZ-induced diabetic mice. **a** Pancreatic histological analysis of proliferating islet cells tagged with BrdU (brown color) within the islets (insulin stain in blue color) was achieved with immunohistochemistry. **b** The number of BrdU-positive cells per section replicating within the islets. **c** Islet area was significantly increased in the IPR group compared to others and **d** islet per section also adopted an identical trend. **e**, **f** The insulin content measured with ELISA in the pancreas and blood of their respective groups. STZ, streptozotocin; IPR, intrapancreatic route; BrdU, bromodeoxyuridine; IVR, intravenous route; no STZ and no MSC, control. Data represent the mean ± SEM, *n* = 6, *p ≤ 0.05

Cell replication rates were studied by injecting BrdU on three sequential days at the end of the experiment. The pancreases were retrieved and scrutinized by immunohistochemistry. Proliferating cells were identified as brown spots labeled with BrdU within the islets. They were co-stained with an anti-insulin antibody processed in blue color as displayed in Fig. 5a. An increased frequency of brown spots indicating replicating islet cells, both insulin positive and non-insulin positive per section, was detected in the IPR (2.25 ± 1.65) compared with STZ (0.12 ± 0.40, *p* ≤ 0.001), IVR (1.37 ± 1.35, *p* ≤ 0.005), and control (0.35 ± 0.73, *p* ≤ 0.001) groups as described in Fig. 5b. Interestingly, not all BrdU-positive cells stained positive for insulin. However, none of the BrdU-positive cells stained positive for glucagon or somatostatin (Supplement Figures S2, S3, S4).

A similar observation was obtained when areas of islet sections [IPR (2247.6 ± 143.2 μm^2^) versus STZ (1546.8 ± 65.7 μm^2^), IVR (1722.6 ± 65.6 μm^2^), and non-diabetic control (4250.4 ± 260.4 μm^2^)] and the number of islets per section [IPR (4.8 ± 2.8) were compared with STZ (1.25 ± 0.96, *p* ≤ 0.001) and IVR (2.6 ± 1.05, *p* ≤ 0.002)] (Fig. 5c, d). In addition, mean insulin content in the blood and pancreas showed a non-significant increase in the sequence STZ, IVR, and IPR (Fig. 5e, f). A statistical discrepancy of total pancreatic insulin content was detected when comparing STZ (9.2 ± 7.6 ng insulin/mg of protein) and IPR (32.17 ± 7.6 ng insulin/mg of protein; *p* ≤ 0.019).

### ADMSC operate through immune modulation, growth factors, and the ERK-DLK1 pathway

**Fig. 6:**
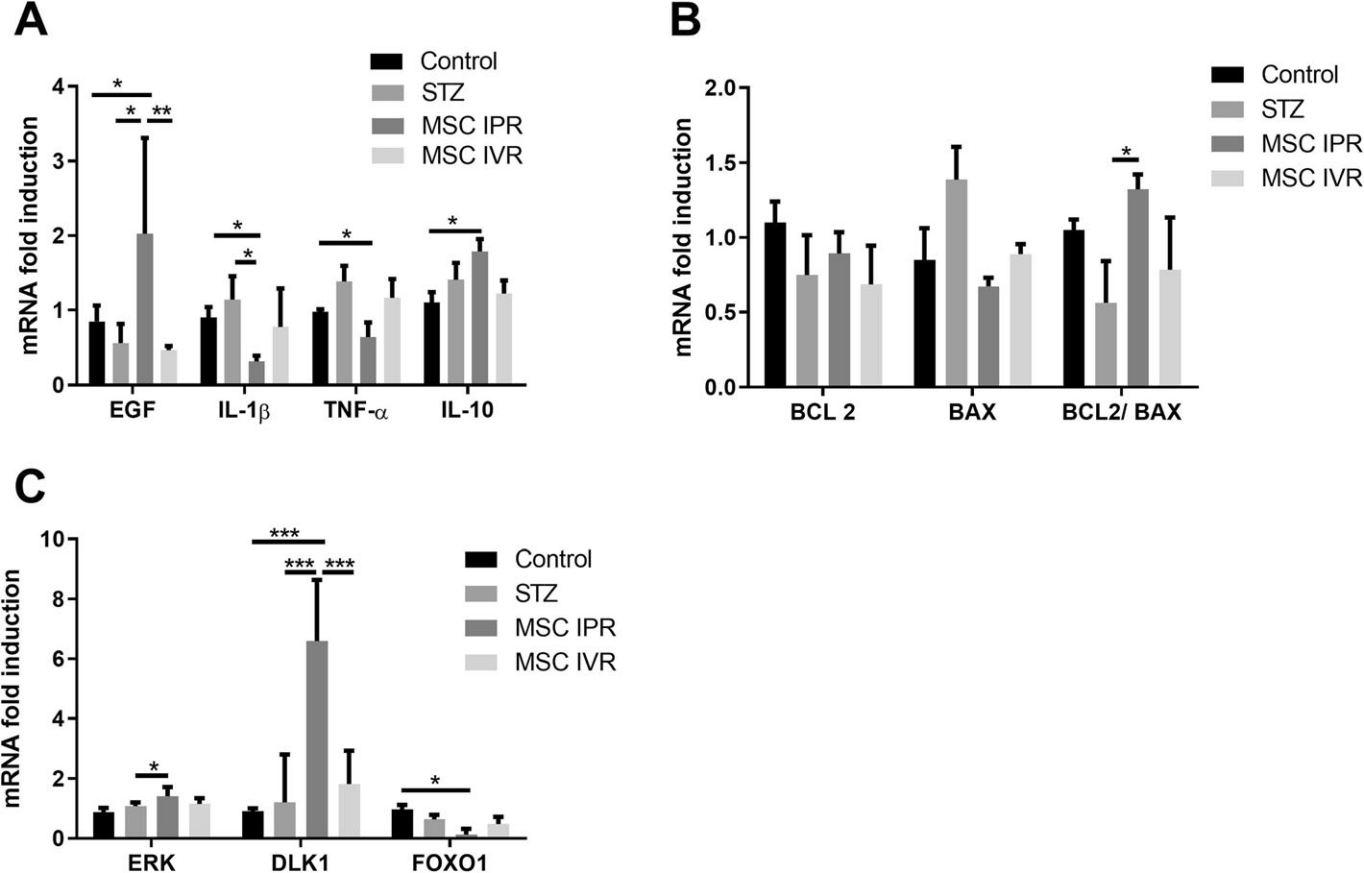
Protective effect of ADMSC in STZ-induced diabetic mice through growth factors, immunomodulation, and the pancreatic DLK1-ERK-FoxO1 signaling cascade. **a** Growth factor (EGF) and pro- and anti-inflammatory transcripts (IL-1β, TNF-α, and IL-10) were measured at the end of the experiment. A higher EGF expression and anti-inflammatory equilibrium was facilitated by ADMSC. **b** Decreased apoptosis after ADMSC administration through the anti-apoptotic (BCL-2) and BAX (apoptotic) molecule ratio was noticed in the IPR group. **c** Pancreatic gene expression of ERK, FoxO1, and DLK1 was determined with RT-PCR after 30 days to STZ injection in all the groups. STZ, streptozotocin; EGF, epidermal growth factor; FoxO1, forkhead box 1; TNF-α, tumor necrosis factor-alpha; IL-1β, interleukin 1 beta; IL-10, interleukin-10; BCL-2, B cell lymphoma 2; BAX, BCL-2-associated X protein; DLK1, delta-like non-canonical notch ligand 1; ERK, extracellular signal-regulated kinases; IPR, intrapancreatic route; IVR, intravenous route; no STZ and no MSC, control. Data show the mean ± SEM, *n* = 3 or 4. Value considered significant at *p* ≤ 0.05, **p < 0.01, and ***p ≤ 0.001

Transcripts of growth factors VEGF, IGF-1, HGF, b-FGF, and EGF in residual pancreatic tissue were quantitated with RT-PCR. The EGF expression was enhanced with ADMSC transplanted locally compared to systemic administration (IVR; *p* ≤ 0.0019), STZ (*p* ≤ 0.023), and non-diabetic control (*p* ≤ 0.042, Fig. 6a). No noteworthy variation was noticed in the expression of VEGF, IGF-1, HGF, and b-FGF (data not presented). Next, pro- and anti-inflammatory genes were investigated. Interestingly, the anti-inflammatory IL-10 expression was at a maximum in IPR compared to the other groups (*p* ≤ 0.019 versus control, Fig. 6a). Concomitantly, the expression of pro-inflammatory IL-1β and TNF-α was minimal in the IPR group (IL-1β, *p* ≤ 0.020 and TNF-α, *p* ≤ 0.036 compared with control), indicating the restoration of the Th1/Th2 equilibrium.

BAX promotes cell death through the permeabilization of the mitochondrial outer membrane in response to cellular stresses. In contrast, BCL-2 prevents apoptosis by inhibiting the activity of BAX. The ratio of anti-apoptotic BCL-2 and apoptotic BAX gene expression was elevated in MSC-IPR compared to the untreated diabetic STZ group indicating a turn from a pro- to an anti-apoptotic environment by application of MSC (*p* ≤ 0.05, Fig. 6b).

To explore the molecular mechanism mediating the proliferative and protective action of ADMSC, the pancreatic DLK1-ERK-FoxO1 signaling cascade was probed with RT-PCR. ADMSC administered into the pancreas increased the ERK (*p* ≤ 0.041) and decreased FoxO1 (*p* ≤ 0.034) expression as compared to the control group (Fig. 6c). Further, pancreatic DLK1 transcripts were enhanced comparing local (IPR) with systemic administration of ADMSC (IVR; *p* ≤ 0.001), STZ (*p* ≤ 0.001), and the non-diabetic control (*p* ≤ 0.001).

## Discussion

The substitution of β cells for T1D patients is a major therapeutic challenge. Recently, embryonic, induced pluripotent, and mesenchymal stem cells have been employed to replace β cells [28–34]. Administration of MSC is advantageous over other stem cells as they ward off the risk of teratoma formation for the recipient [35–37]. In our study, ADMSC were transplanted subcutaneously into NMRI nude mice and observed for 42 days to exclude tumor formation (data not shown). As per International Society Cell and Gene Therapy, MSC are required to be characterized for their cell surface markers (CD44+, CD105+, CD73+, and CD90+), fibroblastic-like structure, and surface adherence properties [25].

MSC are reported to entangle into the lung’s microvasculature after the systemic administration. However, MSC transplanted along with islets improved sustainability and overall engraftment in patients, but it is still ambiguous whether physical contact between MSC and pancreatic β cell is demanded, or a paracrine effect of soluble factors is sufficient [38]. Therefore, we compared the two diverse routes, i.e., local transplantation with cells injected directly into pancreatic parenchyma, and systemic transplantation with ADMSC administered through the tail vein in STZ-induced diabetic NMRI nude mice. This in vivo model was translated to in vitro culture condition to explore intercellular communication on the molecular level. DC and IDC contact of hTERT-MSC (human) with STZ-damaged MIN6-cells (mouse) was investigated.

Higher viability of STZ-injured MIN6-cells after co-culture with MSC in physical contact (DC) was found. By contrast, IDC failed to sustain an analogous effect to MIN6-cells exposed to 2.0 mM STZ. MSC administered through the IVR route showed a restricted migration towards STZ-damaged pancreatic tissue. Therefore, employing the Boyden chamber, MSC migration was assessed towards STZ-damaged MIN6-cells. The expression of SDF-1 (CXCL12) was increased in STZ-damaged MIN6-cells, suggesting that CXCR4 expressing MSC in both DC and IDC were attracted. After interacting with injured MIN6-cells, the MSC manufactured a higher amount of TIMP-1, IDO1, and VEGF in DC which maintained insulin transcript integrity of *Ins2* in MIN6-cells. These results were in conformity with the literature, for instance, increased SDF-1 expression was observed for 7 days in a mouse model of acute pancreatitis. Moreover, IDO1, VEGF, and TIMP-1 were conferred for tissue maintenance [39–41]. Further, both AKT- and ERK-mediated pathways were activated in DC and IDC, but phosphorylation of ERK was more enhanced in DC as compared to IDC. Both DC and IDC were observed to grant protection of the pancreatic β cells through BCL-2/BAX.

Different routes of MSC transplantation for T1D therapy are still debatable in preclinical and clinical studies. The supreme route should provide foremost regeneration with the lowest side effect [42, 43]. Yaochite et al. claimed a stronger effect of the intrasplenic administration of MSC over the intrapancreatic route [19]. Similarly, the intravenous infusion of human umbilical cord-derived MSC had a greater impact than the intrapancreatic one [15]. However, a recent study revealed the dominance of the intrapancreatic infusion in balancing blood glucose level by reducing Iba1-positive and CD40 cells over the intravenous route [20]. Our study further reinforces this recent outcome and claims the superior antidiabetic effect of intrapancreatic infusion of MSC.

In this study, IPR-ADMSC administration displayed lower mean blood glucose and higher body weight after 10–15 days. No such impact was noted in IVR as discussed in the literature [44]. This could be because of the discrepancy in the murine strains or the initial number of MSC being too low (0.5 × 10^6^). It could be further presumed that after transplantation, the majority of MSC were entrapped into the lungs and failed to reach the damaged pancreas for a salutary effect. In our study, after 30 days, ADMSC-DNA was noticed in the lung, kidney, and pancreas after systemic administration (Figure S1). Murai et al. also pointed out the non-functional outcome of intravenous injection in T1D, which supports our results. Further, both the mouse-health-score follow-up and the survival rate confirmed the higher curative effect of IPR infusion over IVR and STZ groups from diabetic symptoms. Likewise, increased pancreatic weight was recorded, which is amalgamated with accelerated recovery from diabetic stress within the short window of 30 days.

We next addressed the increase in pancreatic islet cell replication after IPR injection. Proliferating pancreatic β cells were reported in the neonatal and fetal stages. This proliferative potential rapidly wanes in adult cells [45]. The IPR-ADMSC administration displayed an increased number in replicating islet cells along with an increased pancreatic islet area compared to control and IVR. Surprisingly, rare but low proliferation was further observed in the control group. Recently, it was reported that an immature β cell niche remained throughout life and acted as a source of insulin-producing β cells [46]. No such effect was observed in diabetic mice that had received STZ without ADMSC. Similarly, pancreatic insulin also featured an analogous pattern.

The MSC secretome was reported to counteract the pro-apoptotic microenvironment in the diabetic pancreas [47–50]. Our experiment conforms with these publications as the EGF and IL-10 genes were up- and pro-inflammatory IL-1β and TNF-α were downregulated in the MSC-IPR group. Similarly, anti-apoptotic signaling with the BCL-2 versus BAX ratio was most pronounced in the IPR as contrasted to the other groups. In vitro studies proposed a role of AKT- and ERK-mediated pathway. Therefore, both were studied, and a predominantly higher expression of ERK was detected in the IPR. Further, the FoxO1 nuclear export has a significant impact on β cell proliferation, and the ERK signaling cascade has the potential to modulate it [51, 52]. The FoxO1 expression was seen downregulated in the IPR group. Further, activated DLK1 was noticed after MSC injection through IPR in T1D NMRI nude mice. In the past, DLK1 was linked with the inhibition of adipogenic differentiation [53]. It further cooperates in MSC differentiation as its overexpression influenced the ERK and FoxO1 signaling cascade [54, 55]. DLK1 expresses during embryonic development in the pancreas and declines rapidly after birth [56]. However, a DLK1 receptor is still unknown [57]. This study provides insight into its signaling cascade and points to the antidiabetic influence of MSC-IPR administration which will be feasible to be employed in T1D treatment.

## Conclusion

**Fig. 7:**
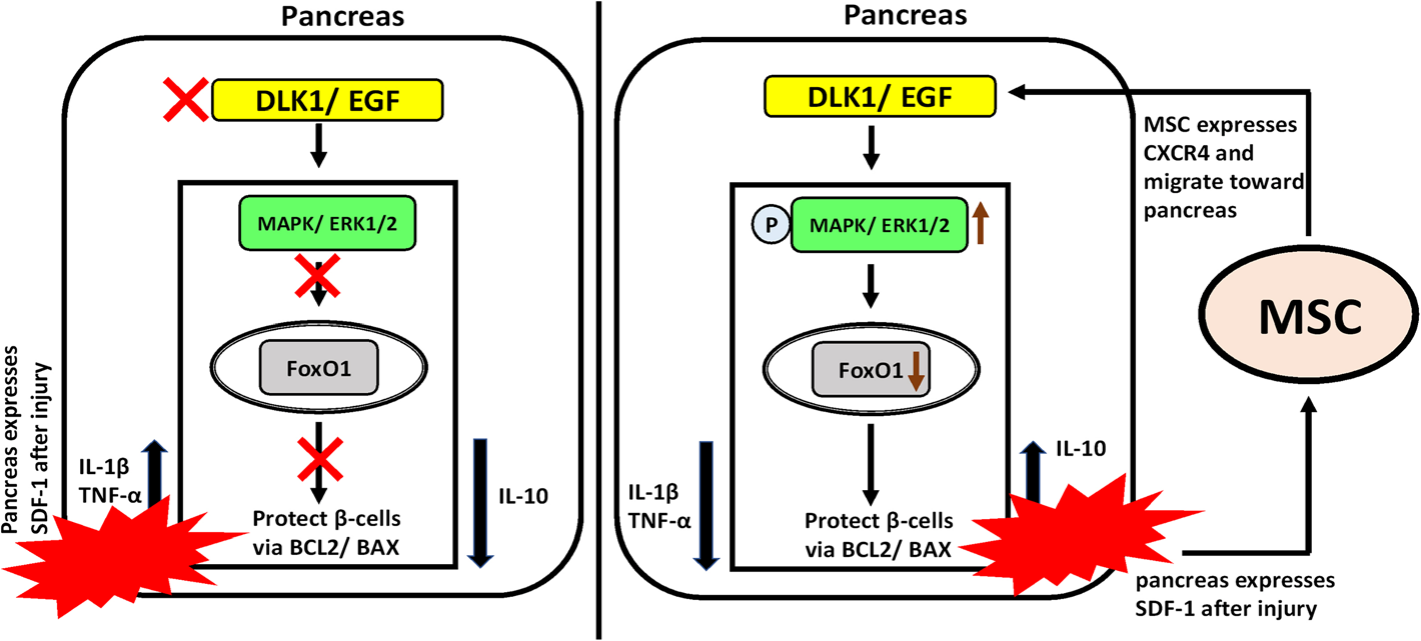
Diagrammatic representation of MSC’s proposed mechanism of protection in STZ-induced diabetic mice. **a** The diabetic pancreas without MSC. **b** IPR administration into the diabetic pancreas stimulates the DLK1-ERK-FoxO1 signaling cascade

Our study delivers further evidence for a superior antidiabetic effect of the local/intrapancreatic (IPR) administration of ADMSC as compared to the systemic/intravenous (IVR) administration in STZ-induced diabetic mice. The local transplantation improved both glycemic control and the animals’ body weight significantly through endogenous pancreatic islet cell replication and EGF release. Further, the IPR route resulted in the regulation of the host’s immune system and the induction of the pancreatic DLK1-ERK-FoxO1 signaling cascade (Fig. 7).

## Supplementary Information

**Additional file 1.**

## Data Availability

All relevant data and material to reproduce the findings are available in the manuscript.

## Abbreviations

IDC: Indirect culture
DC: Direct culture
hTERT-MSC: Human telomerase reverse transcriptase mesenchymal stem cells
DMEM: Dulbecco’s modified Eagle’s medium
CXCR-4: C-X-C chemokine receptor type 4
SDF1: Stromal cell-derived factor 1
IDO1: Indoleamine 2,3-dioxygenase 1
TIMP-1: TIMP metallopeptidase inhibitor 1
VEGF: Vascular endothelial growth factor
ANOVA: Analysis of variance
*Ins2*: Preproinsulin 2
ERK: Extracellular signal-regulated kinases
AKT: Protein kinase B
BCL-2: B cell lymphoma 2
BAX: BCL-2-associated X protein
mRNA: Messenger ribonucleic acid
STZ: Streptozotocin
EGF: Epidermal growth factor
FoxO1: Forkhead box 1
FDA: Fluorescein diacetate
TNF-α: Tumor necrosis factor-alpha
IL-1β: Interleukin 1 beta
IL-10: Interleukin-10
DLK1: Delta-like non-canonical notch ligand 1
IPR: Intrapancreatic route
IVR: Intravenous route
c-DNA: Complementary deoxyribonucleic acid
ESC: Embryonic stem cells
iPSC: Induced pluripotency stem cell
T1D: Type 1 diabetes
β cells: Beta cells
BrdU: Bromodeoxyuridine
ADMSC: Adipose-derived mesenchymal stem cells
RT-PCR: Quantitative real-time polymerase chain reaction
PBS: Phosphate-buffered saline
FBS: Fetal bovine serum
SEM: Standard error of the mean
NMRI: The Naval Medical Research Institute
DMSO: Dimethyl sulfoxide
MTT: 3-(4,5-Dimethylthiazol-2-yl)-2,5-diphenyl tetrazolium bromide
MIN6-cells: Mouse insulinoma 6 cells
