# Supplementary material for: Intrapancreatic MSC transplantation facilitates pancreatic islet regeneration
Source: Stem Cell Res Ther. 2021 Feb 12;12:121. doi: 10.1186/s13287-021-02173-4 (PMC7881671; doi:10.1186/s13287-021-02173-4)
Supplement: Supplementary file 1 — Additional file 1. [file 13287_2021_2173_MOESM1_ESM.docx]

**DNA detection**

At the end of the experiment, several organs such as pancreas, kidney, lung, spleen, liver, and heart were retrieved and subjected to DNA isolation. Lysis buffer (400 mM NaCl, 20 mM Tris-Cl, 1% SDS and 5 mM EDTA) with 0.2 mg/ml Proteinase K at 56 °C for ten hr. Phenol/ chloroform separation was performed followed by DNA precipitation with ethanol. DNA was dissolved in RNase/ DNase free water and measured with NanoDrop spectrophotometer (NanoDrop, USA). PCR was performed for human Alu sequence and product was analyzed at 224 bp on the 1.5 % agarose gel. Human Alu sequence (Forward; 5′-CATGGTGAAACCCCGTCTCTA-3, Reverse; 5′-GCCTCAGCCTCCCGAGTA G-3′)

Further, presence of human genomic DNA or human Alu sequence in mouse organs was quantified. DNA was isolated from 0.5 × 10^6^ human ADMSC and ten-fold serial dilution standard curve was constructed. PCR conditions (initial denaturation; 95 °C for 10 min, denaturation; 95 °C, 15 s for 40 cycles, annealing; 60 °C, 30 min and extension: 60 °C, 1 min and melting curve). Estimation of ADMSC number was determined via Ct value of the standard curve.

**Figure S1**: **S1.1**; Human DNA detection after 30 days of ADMSC infusion in the diabetic pancreas. 0.5 x 10^6^ ADMSC administered in different groups (IVR and IPR). In control and STZ group, human DNA was not observed. In IPR, human Alu sequence was detected only in pancreas (2/10) and IVR, observed in the kidney (1/10), lung (1/10) and pancreas (1/10). Ladder (-1), positive control; human DNA (0), liver (1), lung (2), spleen (3), kidney (4), heart (5) and pancreas (6). Intravenous route (IVR) and intrapancreatic route (IPR), streptozotocin (STZ), n= 5 or 10. **S1.2**. Further, number of transplanted ADMSC through human Alu sequence was estimated. Post 30 days, organs were retrieved and screened for human Alu sequence. In IVR group (lung represented 105 ADMSC, kidney; 50 ADMSC and pancreas; 80 ADMSC) whereas IPR group represented 311 ADMSC in pancreas 1 and 125 ADMSC in pancreas 2.

**Figures S2.1 to 2.4** represent the co-staining of insulin (blue) + BrdU (brown) within islets after ADMSC administration in STZ-induced diabetic mice.


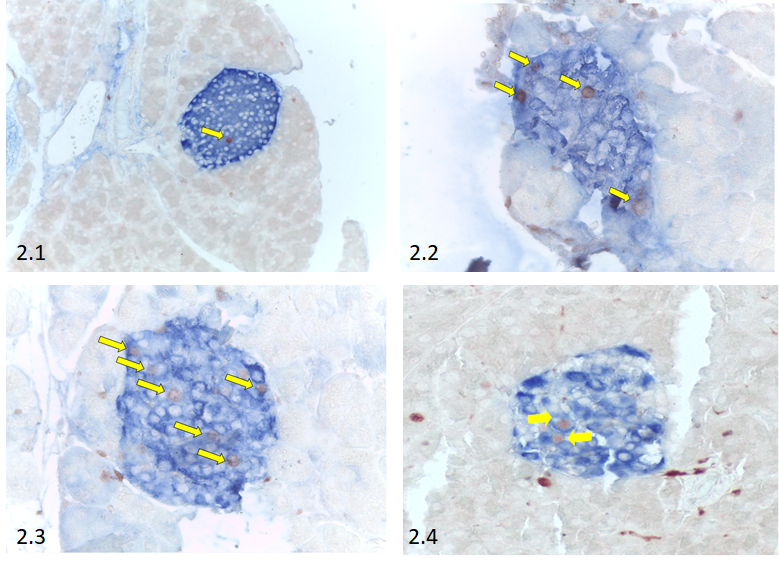


**Figure S2.1 to 2.4:** The morphology of pancreatic islets after ADMSC administration in STZ-induced diabetic mice. Pancreatic histological analysis of proliferating cells tagged with BrdU^+^ (brown color) within the islets (insulin stain in blue color). ADMSC= Adipose tissue derived mesenchymal stem cells

**Figures S3.1 to 3.4** represent the co-staining of glucagon (blue) + BrdU (brown) within the islets after ADMSC administration in STZ-induced diabetic mice.

**
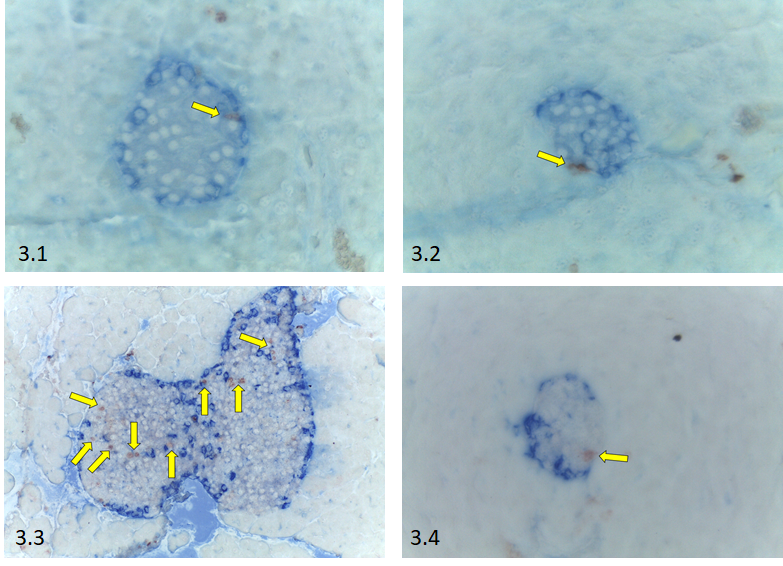
**

**Figure S3.1 to 3.4**: Representative pictures of pancreatic islets after ADMSC administration in STZ-induced diabetic mice. Pancreatic histological analysis of proliferating cells tagged with BrdU^+^ (brown color) within the islets (glucagon stain in blue color).

**Figures S4.1 to 4.4** represent the co-staining of somatostatin (blue) + BrdU (brown) within the islets after ADMSC administration in STZ-induced diabetic mice model.

**
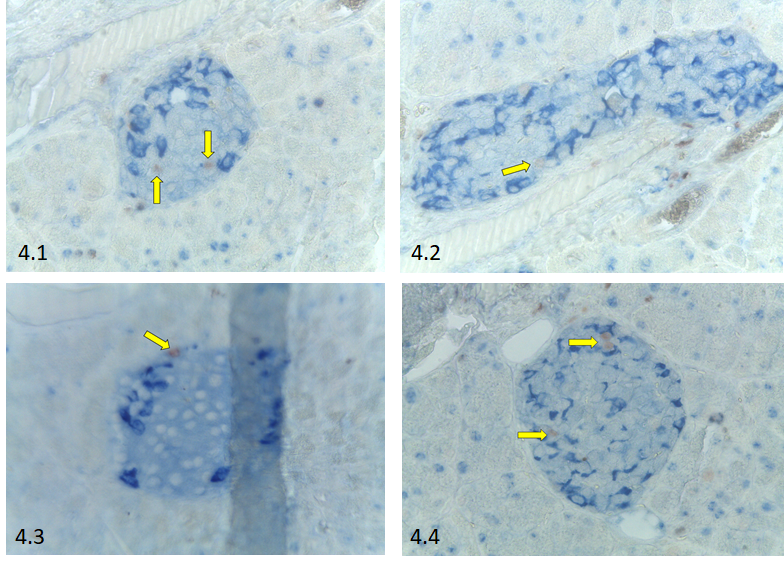
**

**Figure S 4.1 to 4.4**: Demonstrates the images of pancreatic islets after ADMSC administration in STZ-induced diabetic mice. Pancreatic histological analysis of proliferating cells tagged with BrdU^+^ (brown color) within the islets (somatostatin stain in blue color).
